# Remission Achieved in Refractory Advanced Takayasu Arteritis Using Rituximab

**DOI:** 10.1155/2012/406963

**Published:** 2012-10-10

**Authors:** D. Ernst, M. Greer, M. Stoll, D. Meyer-Olson, R. E. Schmidt, T. Witte

**Affiliations:** ^1^Clinic of Immunology and Rheumatology, Medical School Hannover, Carl-Neuberg-Straße 1, 30625 Hannover, Germany; ^2^Department of Pneumology, Medical School Hannover, Carl-Neuberg-Straße 1, 30625 Hannover, Germany

## Abstract

A 25-year-old patient was referred due to subclavian stenosis, identified on echocardiography. She presented with exertional dizziness and dyspnoea. Questioning revealed bilateral arm claudication. Examination demonstrated an absent right ulnar pulse and asymmetrical brachial blood pressure. Bruits were evident over both common carotid arteries. Doppler ultrasound and MRI angiograms revealed occlusion or stenosis in multiple large arteries. Takayasu arteritis (TA) was diagnosed and induction therapy commenced: 1 mg/kg oral prednisolone and 500 mg/m^2^ intravenous cyclophosphamide (CYC). Attempts to reduce prednisolone below 15 mg/d proved impossible due to recurring disease activity. Adjuvant azathioprine 100 mg/d was subsequently added. Several weeks later, the patient was admitted with a left homonymous hemianopia. The culprit lesion in the right carotid artery was surgically managed and the patient discharged on azathioprine 150 mg/d and prednisolone 30 mg/d. Despite this, deteriorating exertional dyspnoea and angina pectoris were reported. Reimaging confirmed new stenosis in the right pulmonary artery. Surgical treatment proved infeasible. Given evidence of refractory disease activity on maximal standard therapy, we initiated rituximab, based on recently reported B-cell activity in TA.

## 1. History

A 25-year-old ICU nurse was referred for further assessment of a subclavian artery stenosis, recently diagnosed on echocardiography. This investigation was performed as part of cardiology assessment for recent-onset dizziness and dyspnoea with moderate exertion: 3 km cycling on level terrain. Heart catheterisation excluded relevant coronary artery disease. With exception of a successfully treated episode of optic neuritis in the right eye 7 years previously, there was no significant past medical history. Investigations at that time had focused on excluding multiple sclerosis, but a small right-sided parietal infarct was noted in the cerebral MRI. Following steroid treatment, the visual disturbance resolved completely. No further investigation or treatment regarding the infarct was performed at that time. The patient had never smoked and had not been taking any regular medication.

## 2. Examination

On general inspection, the patient appeared unremarkable; height: 164 cm and weight: 57.4 kg. Significant clinical findings included an absent right ulnar pulse. Asymmetrical blood pressure measurements were noted: left 88/64 mmHg and right 74/48 mmHg. Auscultation revealed a loud systolic murmur throughout the praecordium, along with bruits over the carotid, subclavian, and femoral arteries bilaterally and the left axillary artery. Skin and joint examinations were entirely unremarkable. There was no clinical suggestion for significant lymphadenopathy. Neurological examination was completely normal.

## 3. Investigations

Routine blood investigations revealed a microcytic anaemia along with raised ESR and CRP at 36 mm/h and 45 mg/L, respectively. Renal function was normal. The ANA titre was minimally elevated at 1 : 320. Other immunological indices including ANCA and cryoglobulins were normal.

**Figure 1:**
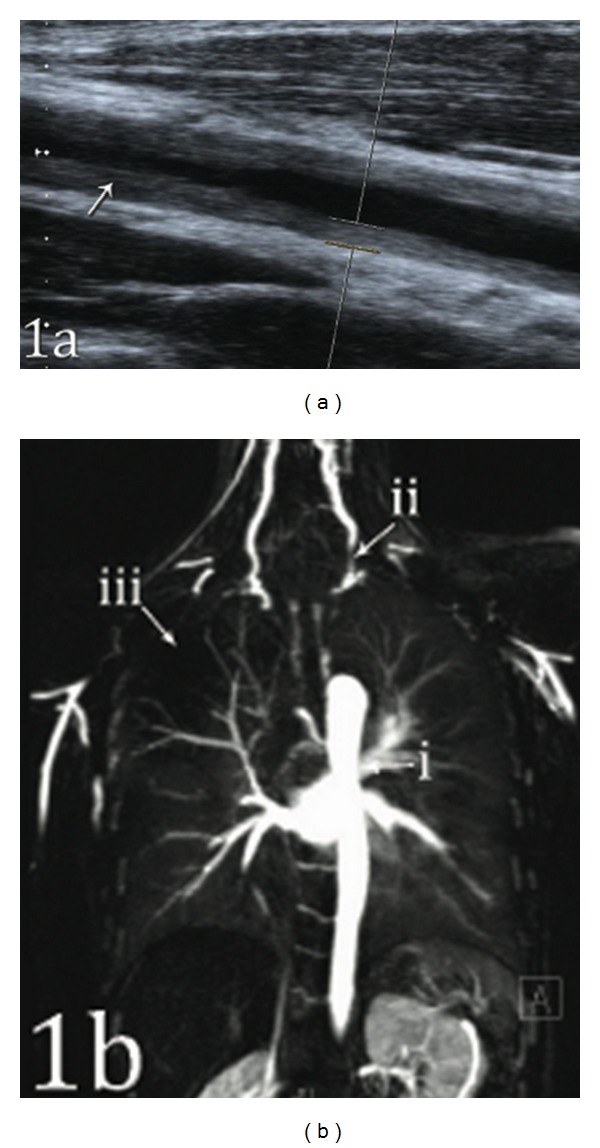
(a) Doppler ultrasound of the left common carotid artery demonstrating marked wall thickening with subsequent loss of lumen inkeeping with stenosis (arrow). (b) MR angiothorax/neck exhibiting widespread pathological changes in the arterial vessels: narrowing of the thoracic aorta (i), stenosis of the left carotid artery (ii), and bilateral occlusion of the subclavian arteries (iii), with collateral distal filling via the intercostal arteries.

To further assess the degree and distribution of arterial stenoses, numerous Doppler ultrasound scans were performed. Carotid Dopplers revealed high-grade stenoses at the bifurcation of the left common carotid artery (Figure 1(a)) and medium grade at the bifurcation of the right common carotid artery. Further assessment demonstrated significant wall thickening in both subclavian arteries, with diminished flow on the left.

In the abdomen, a high-grade stenosis of the right renal artery was observed. In both dorsal pedal arteries, an occlusion pressure in excess of 200 mmHg was recorded.

Given these findings, MR angiography of the head, neck, and thorax (Figure 1(b)) was performed, revealing both acute inflammation and extensive established disease: complete occlusion of the left subclavian and both axillary arteries with good collateralisation. Numerous short high-grade stenoses of the left common carotid artery with complete occlusion of the ipsilateral external carotid artery were seen. Minimal wall thickening with patchy contrast enhancement was observed in the thoracic aorta. A PET-CT scan was considered, but the patient declined this investigation due to the required radiation exposure.

## 4. Treatment and Clinical Course

**Figure 2:**
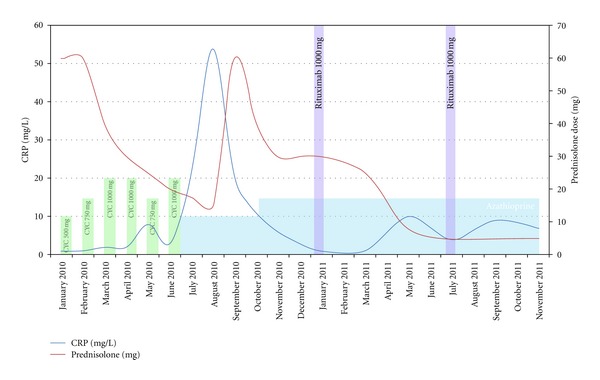
Treatment timeline showing the response of systemic inflammatory markers to different immunosuppressive therapies during the course of treatment. CRP: C-reactive protein; CYC: cyclophosphamide.

Given that all ACR criteria for Takayasu arteritis were fulfilled [1], aggressive induction therapy in accordance with the current EULAR/EUVAS recommendations [2] was commenced: 1 mg/kg oral prednisolone (initially 60 mg, with subsequent tapering—see Figure 2). Due to the disappointing CRP response in the ensuing weeks, adjuvant treatment with intravenous cyclophosphamide in 4 weekly intervals was added: initially 500 mg/m^2^; 2nd dose 750 mg/m^2^ with subsequent courses 1000 mg/m^2^. In total 6 cycles of cyclophosphamide were administered. Thereafter maintenance treatment with azathioprine 100 mg/d was added to 15 mg/d prednisolone. Further attempts to reduce steroid dose below 15 mg/d under maintenance therapy were accompanied by elevated inflammatory indices. Additionally, aggressive antihypertensive therapy consisting of enalapril, amlodipine, and diuretic, along with aspirin and osteoporosis prophylaxis, was commenced.

Six weeks later, the patient presented to her local hospital with acute visual problems. Neurological assessment confirmed a left-sided homonymous hemianopia and motor dysphasia. Intracerebral haemorrhage was excluded. Given simultaneous deterioration in CRP, a vasculitic exacerbation was considered the most likely explanation and high-dose steroids (1000 mg/d IV SDH for 3 days, thereafter a further oral taper commencing with 60 mg/d) were administered. A rapid improvement in CRP and complete resolution of the neurological symptoms were observed. Given the advanced nature of the carotid involvement, an elective left-sided aortocarotid bypass was performed 1 month later. Directly after surgery, a right-sided hemiparesis and new-onset global dysphasia were diagnosed. An emergency MR brain confirmed minor intracerebral bleeding in the brainstem, believed to have resulted from an acute hypertensive crisis in the perioperative phase. Following multidisciplinary discussion, best supportive care was initiated including 12 weeks of intensive physiotherapy in a rehabilitation centre.

Despite ongoing maintenance treatment with azathioprine 150 mg/d and prednisolone 30 mg/d, the patient developed dyspnoea and angina pectoris on exertion (NYHA 3), leading to transfer back to our care from the rehabilitation centre. Repeat MR staging excluded coronary artery involvement but high-grade stenosis of the right pulmonary artery was observed. Following review by colleagues in thoracic surgery, the patient's perioperative risk was deemed unacceptably high due to her poor clinical condition.

Given the failure to achieve satisfactory disease remission despite aggressive immunosuppression and in the absence of other established treatment modalities, the current literature was reviewed and discussed with the patient. Given emerging evidence for B-cell involvement and the efficacy of rituximab [3], this medication was selected as adjuvant therapy and administered per standard rheumatoid arthritis protocol: 1000 mg on D1 and D15, repeated in 6 monthly intervals. Four weeks after the first cycle, the prednisolone dose was successfully reduced. Proceeding in 5 mg/month dose reductions, no evidence of relapse was observed. For the past 12 months, the patient has remained in clinical remission with a maintenance dose of 5 mg/d. Along with the stable inflammatory indices, there has been a steady improvement in clinical and functional statuses: full resolution of the neurological deficit, minimal exertional symptoms: NYHA 2, 3 flights of stairs without a pause, and a return to full-time employment. Repeat MR staging 14 months after commencing rituximab confirmed that there was no indication of active vasculitis and that no further stenoses had occurred.

## 5. Discussion

Our case demonstrates a further example of rituximab-induced sustained remission in Takayasu arteritis, refractory to standard therapy. Unequivocal supporting evidence was demonstrated in terms of CRP and steroid requirement, stabilisation of the MR findings, and a remarkable improvement in both clinical and functional statuses. It once again highlights the dilemma of treating refractory TA, a problem that previous studies have identified in approximately 23% of patients [4]. In recent months, case reports and small retrospective studies have emerged proposing various biological therapies currently licensed for other rheumatological diseases as possible treatment options [3, 5, 6].

Given the good correlation between CRP and clinical course in this patient, we were reluctant to sacrifice this through the use of the IL-6 receptor antagonist tocilizumab [6]. The most data clearly exists for infliximab [6]. We however preferred to use rituximab in our patient for the following two reasons.

1. There was accompanying immunological data implicating B-cell involvement in active TA reported by Hoyer et al. [3]. They demonstrated raised plasmablast levels, leading to increased expression of CD19/CD20/CD27high/HLA-DR^+^ B cells in peripheral blood during acute exacerbations [3].
2. Using protein macroarrays and later on ELISA, we identified autoantibodies against ferritin in the majority of our TA patients (manuscript submitted) including this patient and in almost all our patients with active giant cell arteritis [7]. The presence of these autoantibodies suggests a role for B cells in TA.

Our experience in this case added further evidence to a possible role for biological therapies in TA. Given the rare nature of the disease, numbers treated in individual centres will remain small, making meaningful prospective randomised studies difficult. We therefore propose the establishment of a registry of such patients, incorporating standardised disease staging, markers of activity and functional status to help guide future management in these patients.
